# Neural Androgen Receptors Modulate Gene Expression and Social Recognition But Not Social Investigation

**DOI:** 10.3389/fnbeh.2016.00041

**Published:** 2016-03-16

**Authors:** Sara A. Karlsson, Erik Studer, Petronella Kettunen, Lars Westberg

**Affiliations:** ^1^Department of Pharmacology, Institute of Neuroscience and Physiology, Sahlgrenska Academy, University of GothenburgGothenburg, Sweden; ^2^Department of Psychiatry and Neurochemistry, Institute of Neuroscience and Physiology, Sahlgrenska Academy, University of GothenburgGothenburg, Sweden

**Keywords:** social behavior, memory, three-chambered apparatus test, estrogen, knock-out, sexual dimorphism, autism

## Abstract

The role of sex and androgen receptors (ARs) for social preference and social memory is rather unknown. In this study of mice we compared males, females and males lacking ARs specifically in the nervous system, AR^NesDel^, with respect to social preference, assessed with the three-chambered apparatus test, and social recognition, assessed with the social discrimination procedure. In the social discrimination test we also evaluated the tentative importance of the sex of the stimulus animal. Novel object recognition and olfaction were investigated to complement the results from the social tests. Gene expression analysis was performed to reveal molecules involved in the effects of sex and androgens on social behaviors. All three test groups showed social preference in the three-chambered apparatus test. In both social tests an AR-independent sexual dimorphism was seen in the persistence of social investigation of female conspecifics, whereas the social interest toward male stimuli mice was similar in all groups. Male and female controls recognized conspecifics independent of their sex, whereas AR^NesDel^ males recognized female but not male stimuli mice. Moreover, the non-social behaviors were not affected by AR deficiency. The gene expression analyses of hypothalamus and amygdala indicated that *Oxtr*, *Cd38*, *Esr1*, *Cyp19a1*, *Ucn3*, *Crh*, and *Gtf2i* were differentially expressed between the three groups. In conclusion, our results suggest that ARs are required for recognition of male but not female conspecifics, while being dispensable for social investigation toward both sexes. In addition, the AR seems to regulate genes related to oxytocin, estrogen and William’s syndrome.

## Introduction

It is well established that testosterone is crucial for sexual dimorphisms in rodents as well as other vertebrates regarding social behaviors, such as aggression, mating behaviors and parental care. The behavioral effects of testosterone are mediated by actions of androgen receptors (ARs), and after aromatization of testosterone to 17-beta-estradiol, by estrogen receptors (ERs). These receptors are expressed in specific nuclei of the hypothalamus, amygdala and related regions. Male rodents lacking either their gonads, ARs or ERs display substantially decreased aggression and sexual behaviors (Ogawa et al., 1997; Sato et al., 2004; Pfaff et al., 2005; Zuloaga et al., 2008b; Raskin et al., 2009, 2012; Juntti et al., 2010; Marie-Luce et al., 2013; Studer et al., 2015). Furthermore, some studies show sex differences in duration of social investigation (Johnston and File, 1991; Holmes et al., 2011; Dumais et al., 2013), which probably are mediated by testosterone (Thor, 1980; Thor et al., 1982; Tejada and Rissman, 2012). Although it is less clear to what extent male and female rodents differ with respect to social recognition (Ferguson et al., 2002; Holmes et al., 2011; Veenema et al., 2012), activation of ERs is known to improve social recognition in mice (Choleris et al., 2003; Tang et al., 2005; Pierman et al., 2008; Sanchez-Andrade and Kendrick, 2011). The knowledge about the role of brain ARs for social investigation, social preference and social recognition is, however, remarkably sparse.

Numerous molecules have been shown to modulate social recognition and social investigation in rodents, including oxytocin (Ferguson et al., 2000; Choleris et al., 2006), vasopressin, (Dantzer et al., 1987, 1988), nitric oxide (Trainor et al., 2007; Wass et al., 2009), dopamine (Makridakis et al., 2000), regulators of the stress response (Deussing et al., 2010; Kasahara et al., 2011), as well as molecules causing social deficits in humans (Samaco et al., 2008; Sakurai et al., 2011; Smith et al., 2011). Although there is strong support for sex hormones playing a crucial role in the regulation of expression of many of these molecules, few studies have so far investigated if they are sexually dimorphic or influenced by ARs.

The main goal of this study was to investigate how sex and ARs modulate sociability, measured with the three-chambered apparatus test (Moy et al., 2004), and social recognition, measured with the social discrimination procedure (Engelmann et al., 2011). To this end, we compared male and female control mice as well as males lacking ARs specifically in the nervous system. Furthermore, we evaluated to what extent the sex of the stimulus animal moderate social investigation and recognition in the social discrimination test. Finally, in order to reveal molecules involved in the effects of sex and androgens on social behaviors we compared the expression levels of genes known to effect sociability and social memory between the three groups of animals.

## Material And Methods

### Animals

#### Conditional CNS-Specific Androgen Receptor Knock-out Mice

Animals, in both the behavioral and gene expression experiments, included conditional AR knock-out (AR^NesDel^) male mice as well as female and male littermate controls. Generation of AR^NesDel^ has been described in detail elsewhere (Raskin et al., 2009). In brief, male C57Bl/6 mice expressing CRE driven by the neuronal *Nestin* promoter (Tronche et al., 1999) were mated with female mice carrying *LoxP* sites flanking the second exon of the AR gene (De Gendt et al., 2004), which had been backcrossed into the C57Bl/6 background for at least six generations prior to the arrival at our laboratory (Bourghardt et al., 2010). Genotypes were confirmed with PCR. In the behavioral experiments 9 AR^NesDel^ males, 26 male (8 wildtype, 9 AR^flox^, 9 NesCre^+/-^) and 10 female (AR^flox+/-^) control mice, in the age range of 5–10 months old, were used. The gene expression experiment comprised 12 AR^NesDel^ males, 8 male (1 wildtype, 1 AR^flox^, 6 NesCre^+/-^) and 9 female (8 AR^flox+/-^ and 1 wildtype) control mice, all in the age range of 4–5 months.

#### Estrus Cycle

To determine the estrus cycle for the female controls, vaginal smears were taken directly after both the three-chambered test and the social recognition test. The smears were obtained by gently flushing the vagina with a PBS solution (Life Technologies, USA), while care was taken not to induce pseudo-pregnancy (Sinha et al., 1978). Smears were counterstained with tryptophan blue to aid cell counting by light microscopy (Nikon eclipse 90i, Nikon Instruments Europe). Determination of the estrus cycle stages (metestrus, estrus, diestrus, and proestrus) was done according to Byers et al. (2012). For the analyses the intact females were divided into two equally sized groups: “estrus” (proestrus and estrus) and “non-estrus” (metestrus and diestrus).

#### Stimulus Mice

To evaluate the importance of sex of the stimulus animals in the social discrimination paradigm, we used both female and male *C57Bl/6N* mice (n = 15 of each sex). Females were gonadectomized (GDX) 2 weeks prior to testing, at the age of 4–5 months. Mice were anesthetized using a 3:12 vol/vol mixture of ketamine (Ketalar 10 mg/ml, Pfizer) and xylazine (Rompun Vet, 20 mg/ml, Bayer Animal Health) and gonadectomy was performed via a midline incision and the ovaries were removed from the female mice. All effort was made to prevent any suffering of the animals during surgery. The stimulus animals were allowed to recover in groups for 4 weeks, until being single-housed 1 week prior to testing. During the social tests the stimulus animals were presented to the test animals in wire corrals (Galaxy pencil cup; Moy et al., 2004). To avoid disturbing behaviors throughout the test, like aberrant bar biting caused by unnecessary stress, the stimulus mice were habituated to the wire corrals during 15 min for 2 days prior to the test start.

#### Experimental Conditions

All mice were held in a conventional animal facility with 12 h light/12 h dark cycle (lights on at 6.00 AM), given *ad libitum* access to food and water. One week before initiating the social experiments, the test animals were habituated to new standard cages during approximately 10 min for 5 days. At least 45 min before tests were started, all mice used in the tests were transported from the housing room to the animal testing area. For all experiments, the test room had an illumination of ∼20 lux and was kept free from strong smells and sounds. Behavioral experiments were performed between 9 am and 5 pm. The corrals, objects and the three-chambered apparatus were cleaned with 70% ethanol followed by water before and between the tests. All procedures were subjected to approval by the Ethical Committee on Animal Experiments, Gothenburg, Sweden (permit number 323-2010 and 313-2011) and performed accordingly.

### Behavioral Experiments

#### Three-Chambered Social Approach Test

The three-chambered social approach test (Moy et al., 2004) was used to assess persistence of social investigation as well as social preference, measured as preference for a novel conspecific vs. an object. After acclimatization to the testing room, focal mice were allowed to freely explore the three-chambered apparatus and habituate to the test arena for 20 min with two empty corrals placed in each side-chamber. After the habituation period, the focal mouse was led to the middle chamber and the doors to both side-chambers were closed. A stimulus mouse was placed in one of the corrals in one of the side-chambers leaving an empty corral in the other side-chamber. The doors between the chambers were removed and the behavior of the focal individual was recorded for 10 min with an overhead video camera.

#### Social Discrimination Test

The social discrimination test (Macbeth et al., 2009) was used to test the ability to remember an already encountered conspecific, i.e., social memory. The test was initiated with two habituation steps and followed by two collection parts: the social investigation part (sample) and social memory part (choice), separated by an inter-trial interval of 30 min. The test mouse was first allowed to habituate to the test cage (41 cm × 25 cm × 14 cm) for 15 min, and after that, to two wire corrals placed in the arena for 30 min. After the habituation steps one corral was removed from the cage and a stimulus mouse was placed in the remaining corral. The 10 min test session started as soon as the stimulus mouse was introduced to the test subject, and was recorded with an overhead video camera. After the sampling session the test mouse was left in the cage with the two empty corrals for 30 min. The choice session for testing short-term social memory began when the test mouse was presented with both the familiar stimulus mouse (from the sample session), and a novel mouse enclosed in separate corrals, for 5 min. Based on the choice session we calculated a social memory score: time exploring novel mouse/(time exploring novel mouse + time exploring familiar mouse), where a ratio above 0.5 indicates social memory.

#### Novel Object Recognition Test

The novel object recognition (NOR) task (Leger et al., 2013) was used to confirm the presence of object memory. NOR was conducted in a similar way as the social discrimination test: the test mouse was placed in a test cage (41 cm × 25 cm × 14 cm) for 30 min of habituation. In the first part of the test, the sampling session, the test mouse was presented to two similar objects for 5 min. After a 30 min inter-trial interval, object memory was assessed during a 5 min choice session with a familiar object, (same as in sampling session) together with a novel object. The objects used in the test were one triangular, one cubic, one round, and one cylindrical object of different materials. During both test sessions the objects were placed in opposite and symmetrical corners of the arena and both the novel and familiar object was counterbalanced between mice in order to avoid object and place preference effects. Similar to the social memory score, an object memory score was calculated as: time exploring novel object/(time exploring novel object + familiar object) where a ratio above 0.5 indicates object memory. The data from one AR^NesDel^ male was missing due to technical problems with movie acquisition.

#### Olfactory Habituation/Dishabituation Test

Because olfactory cues are crucial in mouse behavior we carried out the olfactory habituation/dishabituation test (Yang and Crawley, 2009) to investigate if the mice could detect and discriminate between different odors. The test mouse was habituated to a new cage containing a clean cotton tip 30 min prior to testing. The test consisted of exposure to different odors in a sequence; each odor was presented on cotton tips with durations of 2 min × 3 repeats: water, non-social odor number 1, non-social odor number 2, followed by a social odor, with a 1 min inter-trial interval. Lemon oil (Sigma–Aldrich, Sweden) and cinnamon oil (AROMA Creative AB, Sweden) were used as non-social odors. The social odor was obtained by swabbing the cotton tip in the bedding of a cage with female mice.

#### Scoring Criteria

The scoring criteria for the social tests are described in Macbeth et al. (2009) and Yang et al. (2011), respectively. In the sociability test both the duration time in each chamber and the sniffing time were recorded. In all tests using stimulus mice, sniffing was scored as the time the test mouse was close to and directed toward the stimulus animal or to any part of the mouse (e.g., the tail) positioned outside of the wire corral, as well as when the nose and forepaws were inserted between the corral bars. In contrast, sniffing directed to the upper and top part of the wire corral, sniffing of feces, bar biting and circulating around the corral without sniffing, did not qualify for scoring. For NOR and olfactory test, the scoring criteria were met when the sniffing occurred approximately 2 cm from the objects or cotton swabs. Scoring these behaviors was performed by one trained observer blind to the status of the test mice.

### Gene Expression

In the gene expression experiment we investigated to what extent the mRNA expression levels of 43 specific genes differed in the amygdala and hypothalamus between male and female mice, as well as males lacking ARs in the nervous system.

#### Tissue Preparation

Mice in the ages of 4–6 months were sacrificed by decapitation; brains were then removed and immediately frozen in liquid nitrogen and stored in -80°C before further dissection of the amygdala and hypothalamus. Brains were placed in a cold mouse brain matrix (Zivic instruments, Pittsburg, PA, USA) and cut in 1 mm sections. Amygdala was dissected out, medially along the optic nerve and laterally toward the perirhinal cortex, from sections corresponding to bregma levels -1.06 to -2.06 (plates 40–48) according to Paxinos brain atlas. Hypothalamus was dissected from two sections corresponding to bregma -0.10 to -2.06 (plates 32–48), ventrally from the anterior commissure and extending laterally to the lateral ventricle.

#### Sample Preparation

For total RNA extraction, the brain tissue samples were homogenized using a TissueLyser II (Qiagen) and RNA extraction was performed using RNeasy Lipid Tissue Mini Kit (Qiagen) according to the manufacturer’s instructions. Concentration and quality of RNA were assessed using a NanoDrop (Thermal Scientific, Odessa, TX, USA) and the RNA concentration was adjusted in all samples prior to cDNA synthesis. The total RNA (250 ng per sample) was reverse-transcribed into cDNA in a 20 μl reaction using the high-capacity cDNA reverse transcript kit (Applied Biosystems, Life technologies) as per manufacturer’s instructions.

#### Gene Expression

To measure the gene expression levels of our genes of interest, we used a quantitative real-time PCR (qRT-PCR) analysis that was conducted on an ABI 7900HT Thermocycler (Applied Biosystems, Life Technologies) using 384-well TaqMan^®^ Custom Array microfluidic cards (Applied Biosystems, Life Technologies). Each port on the TaqMan array was loaded with 250 ng cDNA per sample combined with nuclease-free water and 50 μl reaction mixture using fluorescent probe sequences (TaqMan Gene Expression Master Mix, Applied Biosystems, Life Technologies). In **Table 1** all target and control genes are listed. The most stable pair out of five endogenous controls was calculated using DataAssist^TM^ software (Life Technologies). The selected control genes for amygdala was *Gapdh* and *Ppia* and for hypothalamus *Gapdh* and *Actb.* To calculate differences in gene expression we used the comparative *C*_T_ method, described in detail previously (Livak and Schmittgen, 2001), with the male control group set as the calibrator. The Δ*C*_T_ values were calculated by subtracting the mean threshold cycle (*C*_T_) of the two endogenous controls and the mean *C*_T_ from the target gene. ΔΔ*C*_T_ was calculated by subtracting the mean Δ*C*_T_ for the male group (calibrator) with the individual Δ*C*_T_ for each subject. Data is presented as fold change, calculated as 2^-ΔΔCT^. For each triplicate the standard deviation was calculated within the triplicate, and if the standard deviation were above 0.4 the value from that individual was removed and not further analyzed. The final number of samples in each group is stated in **Figures 6**–**8**.

**Table 1:** Names and assay numbers of the investigated genes.

Gene name	Protein name	Assay number
**Control genes**		
*18S*	Eukaryotic 18S rRNA	Hs99999901_s1
*Actb*	Actin, beta	Mm02619580_g1
*Gapdh*	Glyceraldehyde-3-phosphate dehydrogenase	Mm99999915_g1
*Hmbs*	Hydroxymethylbilane synthase	Mm01143545_m1
*Ppia*	Peptidylprolyl isomerase A	Mm02342429_g1
**Oxytocin and vasopressin**		
*Arnt2*	Aryl hydrocarbon receptor nuclear translocator 2	Mm00476009_m1
*Avp*	Arginine vasopressin	Mm01271704_m1
*Avpr1a*	Arginine vasopressin receptor 1A	Mm00444092_m1
*Avpr1b*	Arginine vasopressin receptor 1B	Mm01700416_m1
*Cd38*	CD38 antigen	Mm00483146_m1
*Nos1*	Nitric oxide synthase 1, neuronal	Mm00435175_m1
*Oxt*	Oxytocin	Mm01329577_g1
*Oxtr*	Oxytocin receptor	Mm01182684_m1
*Pam*	Peptidylglycine alpha-amidating monooxygenase	Mm01293044_m1
*Prkcd*	Protein kinase C, delta	Mm00440891_m1
*Sim1*	Single-minded homolog 1	Mm00441390_m1
**Sex steroids**		
*Akr1c14*	Aldo-keto reductase family 1, member C14	Mm00506338_m1
*Ar*	Androgen receptor	Mm00442688_m1
*Crebbp*	CREB binding protein	Mm01342452_m1
*Cyp19a1*	Cytochrome P450, family 19, subfamily a, polypeptide 1	Mm00484049_m1
*Esr1*	Estrogen receptor 1 (alpha)	Mm00433149_m1
*Esr2*	Estrogen receptor 2 (beta)	Mm00599821_m1
*Gper*	G protein-coupled estrogen receptor 1	Mm01194815_m1
*Ncoa1*	Nuclear receptor coactivator 1	Mm00447958_m1
*Ncoa2*	Nuclear receptor coactivator 2	Mm00500749_m1
*Ncoa7*	Nuclear receptor coactivator 7	Mm00552797_m1
*Ncor1*	Nuclear receptor co-repressor 1	Mm00448681_m1
*Ncor2*	Nuclear receptor co-repressor 2	Mm00448796_m1
*Pgr*	Progesterone receptor	Mm00435628_m1
*Srd5a1*	Steroid 5 alpha-reductase 1	Mm00614213_m1
*Srd5a2*	Steroid 5 alpha-reductase 2	Mm00446421_m1
**Stress regulators**		
*Crh*	Corticotropin releasing hormone	Mm01293920_s1
*Crhr1*	Corticotropin releasing hormone receptor 1	Mm00432670_m1
*Crhr2*	Corticotropin releasing hormone receptor 2	Mm00438303_m1
*Nr3c1*	Nuclear receptor subfamily 3, group C, member 1	Mm00433832_m1
*Ucn*	Urocortin	Mm00445261_m1
*Ucn2*	Urocortin 2	Mm01227928_s1
*Ucn3*	Urocortin 3	Mm00453206_s1
**Dopamine**		
*Comt1*	Catechol-O-methyltransferase	Mm00514377_m1
*Drd1a*	Dopamine receptor D1A	Mm01353211_m1
*Drd2*	Dopamine receptor D2	Mm00438545_m1
*Drd3*	Dopamine receptor D3	Mm00432887_m1
*Drd4*	Dopamine receptor D4	Mm00432893_m1
*Drd5*	Dopamine receptor D5	Mm00658653_s1
*Th*	Tyrosine hydroxylase	Mm00447557_m1
**Social deficits**		
*Gtf2i*	General transcription factor II	Mm00494826_m1
*Mecp2*	Methyl CpG binding protein 2	Mm00465017_m1
*Ube3a*	Ubiquitin protein ligase E3A	Mm00839910_m1


### Statistical Analysis

For the behavioral tests statistical associations were estimated using linear mixed model using the MIXED procedure (PROC MIXED) of SAS 9.3 (SAS Institute, Inc., Cary, NC, USA). PROC MIXED is a repeated measurement analysis that allows both fixed and random effects/variables. This model was preferred since it handles missing (random) values and uses maximum-likelihood estimation instead of sums of squares. For the social and object memory scores one-sample *t*-tests with a test value of 0.5 was calculated using SPSS (IBM SPSS Statistics for Windows, Version 19.0, IBM Corp., USA). A *p-*value below 0.05 was considered statistically significant.

The linear mixed model using the MIXED procedure (PROC MIXED) was applied to the Δ*C*_T_ values from the qRT-PCR data to calculate the effect of sex and AR knock-out. The fold change is presented in graphs as percentage up or down regulation relative to the male group. Only associations with *p*-values below 0.05 are discussed in this study and no corrections for multiple testing were performed. An outlier test was performed on the delta Δ*C*_T_ values with Grubbs’ test in Graph Pad Prism 6.

## Results

### Do Sex and ARs Influence Social Preference, Social Investigation and Social Recognition?

**FIGURE 1:**
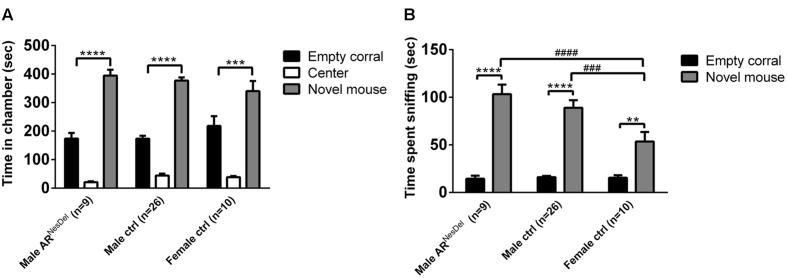
**Sociability measured with the three-chambered apparatus test in AR^NesDel^ males, male controls (wildtype, AR^flox^, NesCre^+/-^), and female controls (ARflox^+/-^). (A)** Amount of time spent in the each chamber during the 10 min test of sociability. **(B)** Amount of time spent sniffing the empty corral or the novel mouse. Bars represent mean ± SEM, ^∗∗^*p* < 0.01, ^∗∗∗^*p* < 0.001, ^∗∗∗∗^*p* < 0.0001 (within-group comparison) and ###*p* < 0.001, ####*p* < 0.0001 (between-group comparison).

Male AR^NesDel^ mice, as well as male and female control mice, were investigated in the three-chambered apparatus in order to determine if sex and/or presence of ARs in the nervous system influenced the sociability (**Figure 1**). All three groups spent more time in the social chamber containing a GDX female stimulus mouse than in the non-social chamber containing an empty corral (within-group comparisons, *p* < 0.001; **Figure 1A**). In addition, all groups spent more time sniffing the female stimulus mice compared to exploring empty corrals (within-group comparison, *p* < 0.01; **Figure 1B**). Results also showed that both AR^NesDel^ and control males spent more time investigating the stimulus mice compared to the female group (*p* < 0.001; **Figure 1B**). When comparing entries between chambers no differences were identified between the three groups (data not shown).

**FIGURE 2:**
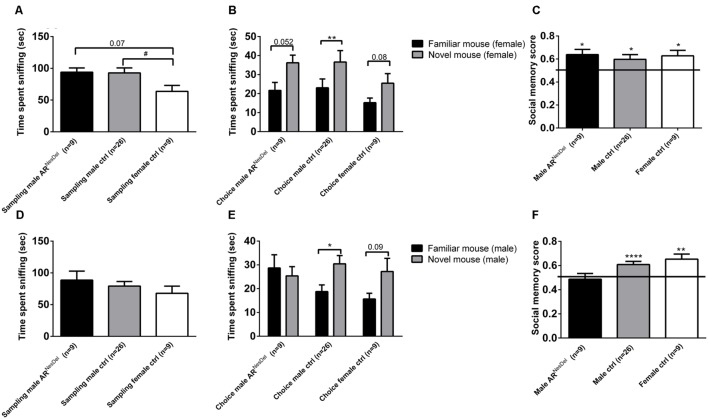
**Social investigation and social recognition measured in the social discrimination paradigm in AR^NesDel^ males, male controls (wildtype, AR^flox^, NesCre^+/-^), and female controls (ARflox^+/-^). (A)** Amount of time spent sniffing female stimulus animals in the sampling phase. **(B)** Social recognition of female stimulus animals. **(C)** Social memory score when presented to female stimulus animals. **(D)** Amount of time spent sniffing male stimulus animals in the sampling phase. **(E)** Social recognition of male stimulus animals. **(F)** Social memory score when presented to male stimulus animals. Bars represent mean ± SEM, ^∗^*p* < 0.05, ^∗∗^*p* < 0.01, ^∗∗∗∗^*p* < 0.0001 (for within-group comparison) and #*p* < 0.05 (for between-group comparison).

The same three groups of mice were investigated in the social discrimination task, both with GDX female and intact male stimuli mice. When presented to stimuli females in the sampling phase, both control and AR^NesDel^ males (*p* = 0.07) were engaged in social investigation for longer time than female controls (**Figure 2A**). When presented to stimulus males all three groups showed similar investigation time in the sampling session (**Figure 2D**). In the second session of the social discrimination test, the choice session, control males and females, but not AR^NesDel^ males, displayed social memory (social memory score > 0.5), when tested with a male stimulus animal (*p* < 0.05; **Figures 2E,F**). In contrast, all three groups showed social memory, when presented to a female stimulus animal (*p* < 0.05; **Figures 2B,C**).

### Does the Estrus Cycle Influence Social Memory or Sociability in Female Mice?

**FIGURE 3:**
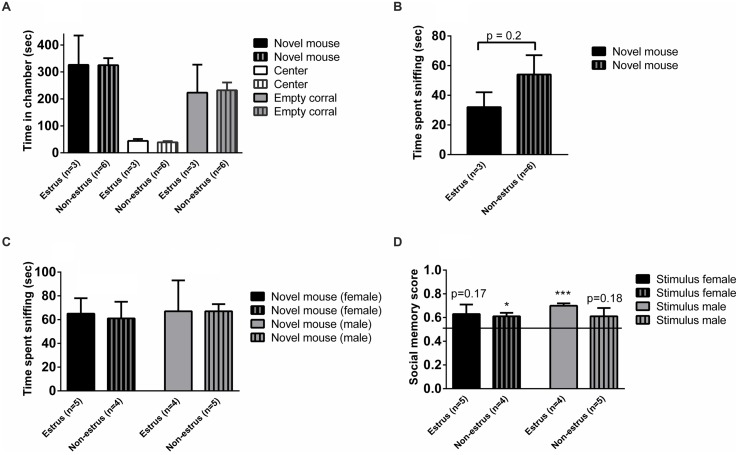
**Sociability, social investigation and social recognition measured in the three-chambered apparatus test or the social discrimination paradigm in estrus and non-estrus female controls (ARflox^+/-^). (A)** Amount of time spent in each chamber and **(B)** sniffing the novel mouse during the 10 min test of sociability in the three-chambered apparatus. **(C)** Amount of time sniffing novel female and male stimulus mice in the sampling phase of the social discrimination paradigm. **(D)** Social memory score when presented to female or male stimulus animals. Bars represent mean ± SEM, ^∗^*p* < 0.05, ^∗∗∗^*p* < 0.001 (for within-group comparison).

The estrus cycle was measured in the control females in order to investigate if the estrus stage may affect social memory or sociability. Females in estrus and non-estrus cycle phases displayed similar chamber times (**Figure 3A**), and exhibited similar duration of sniffing of stimulus animals in the three-chambered apparatus test (*p* = 0.2; **Figure 3B**) as well as in the sampling phase of the social discrimination task, regardless of whether female (*p* = 0.8) or male (*p* = 0.9) stimulus mice were investigated (**Figure 3C**). The social recognition ability seemed to be modulated by estrus phase and sex of the stimulus animals, with the most evident social recognition seen by estrus females memorizing stimulus males (**Figure 3D**). However, the small sample sizes make it difficult to draw firm conclusions from these results.

### Do Sex and Lack of ARs in the Nervous System Influence Novel Object Recognition or Olfaction?

**FIGURE 4:**
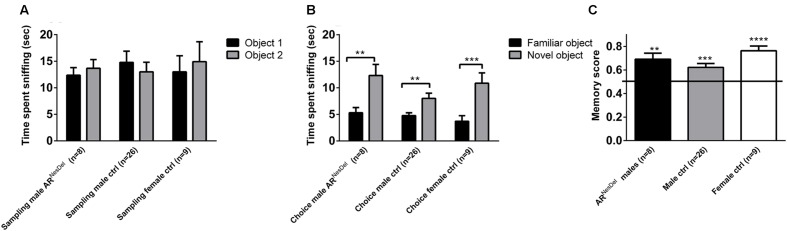
**Novel object recognition (NOR) memory test in AR^NesDel^ males, male controls (wildtype, AR^flox^, NesCre^+/-^), and female controls (ARflox^+/-^). (A)** Amount of time spent sniffing two similar objects. **(B)** Amount of time sniffing a familiar object or a novel. **(C)** Object memory score. Bars represent mean ± SEM, ^∗∗^*p* < 0.01, ^∗∗∗^*p* < 0.001, ^∗∗∗∗^*p* < 0.0001 (for within-group comparison).

**FIGURE 5:**
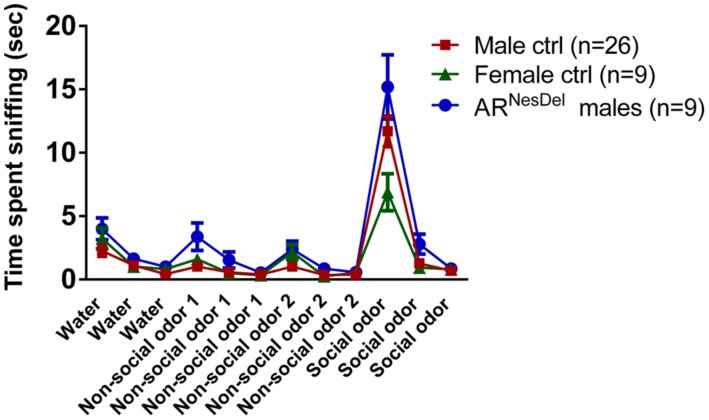
**Odor habituation/dishabituation in AR^NesDel^ males, male controls (wildtype, AR^flox^, NesCre^+/-^), and female controls (ARflox^+/-^).** Data points represent mean ± SEM.

Novel object recognition and odor habituation/dishabituation were investigated to ensure that results from the social tests were not influenced by disturbances in cognitive or sensory functions. In the sampling test of novel object memory where two similar objects were explored, none of the groups showed any preference for objects (**Figure 4A**). In the second part, the choice test, where a familiar object and a novel object were explored, all three groups showed object memory, corresponding to an object memory score above 0.5 (*p* < 0.01; **Figures 4B,C**). In the olfactory habituation/dishabituation test all groups habituated to the odors presented, though the male groups showed a tendency to investigate the social odor for longer durations than the females (**Figure 5**).

### Do Sex and Lack of AR in the Nervous System Influence the Expression of Genes Involved in Social Behavior?

**FIGURE 6:**
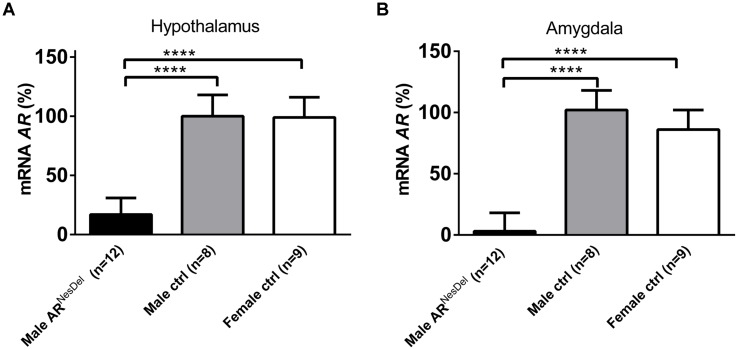
***Ar* mRNA expression levels **(A)** in the hypothalamus and **(B)** in the amygdala of male AR^NesDel^, as well as in male (wildtype, AR^flox^, NesCre^+/-^) and female (wildtype, ARflox^+/-^) controls.** The results are presented as percent (± SEM) in relation to the male control group. ^∗∗∗∗^*p* < 0.0001.

**FIGURE 7:**
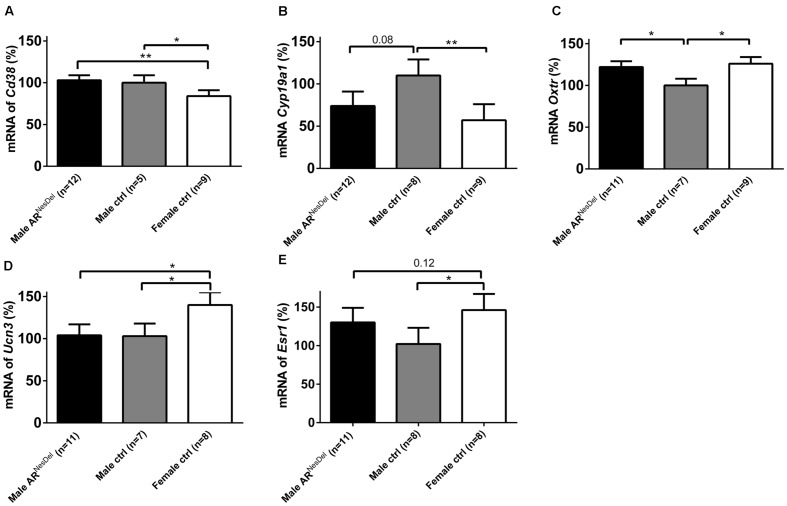
**mRNA expression levels of **(A)***Cd38***(B)***Cyp19a1***(C)***Oxtr***(D)***Ucn3*, and **(E)***Esr1* in the hypothalamus of male AR^NesDel^, as well as in male (wildtype, AR^flox^, NesCre^+/-^) and female (wildtype, ARflox^+/-^) controls.** The results are presented as percent (± SEM) in relation to the male control group. ^∗^*p* < 0.05, ^∗∗^*p* < 0.01.

**FIGURE 8:**
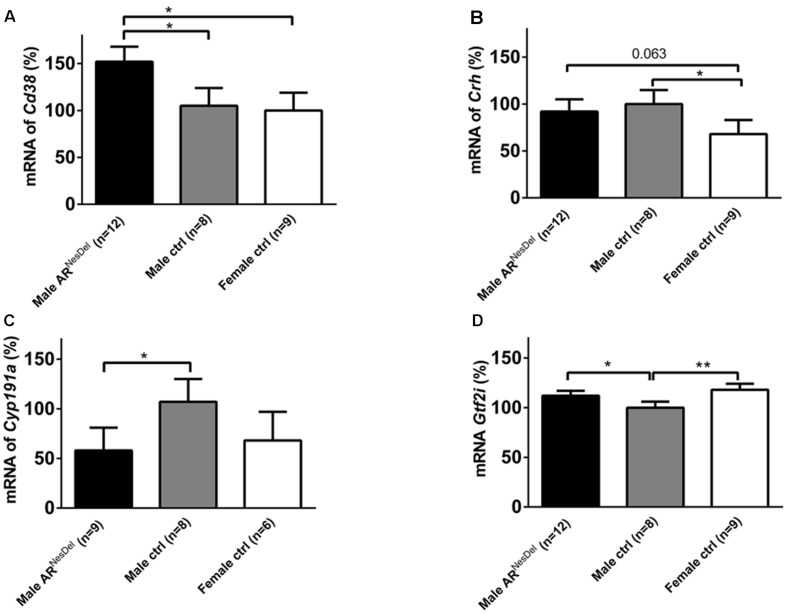
**mRNA expression levels of **(A)***Cd38*, **(B)***Crh*, **(C)***Cyp19a1*, and **(D)***Gtf2i* in the amygdala of male AR^NesDel^, as well as in male (wildtype, AR^flox^, NesCre^+/-^) and female (wildtype, ARflox^+/-^) controls.** The results are presented as percent (± SEM) in relation to the male control group. ^∗^*p* < 0.05, ^∗∗^*p* < 0.01.

Gene expression analysis was performed to investigate if the expression levels of genes of known importance for social behaviors differ between the sexes and/or are modulated by AR. The results from amygdala and hypothalamus of females, males and AR^NesDel^ males confirmed that the expression levels of the AR gene was significantly lower in both brain regions of AR^NesDel^ males compared to both female and male control groups (*p* < 0.0001; **Figure 6**). The overall comparison of expression levels in hypothalamus of the three groups showed significant changes in CD38 antigen (*Cd38*; *p* = 0.01; **Figure 7A**), cytochrome P450, family 19, subfamily A, polypeptide 1 (*Cyp19a1*; *p* = 0.019; **Figure 7B**), oxytocin receptor (*Oxtr*; *p* = 0.035; **Figure 7C**), Urocortin 3 (*Ucn3*; *p* = 0.038; **Figure 7D**), and ERα (*Esr1*; *p* = 0.04; **Figure 7E**). The overall comparison in amygdala showed significant changes in *Cd38* (*p* = 0.019; **Figure 8A**), corticotropin releasing hormone (*Crh*; *p* = 0.046; **Figure 8B**), *Cyp19a1* (*p* = 0.044; **Figure 8C**) and in the general transcription factor II (*Gtf2i*; *p* = 0.024; **Figure 8D**). The results from the *post hoc* analyses are described in **Figures 6**–**8** as well as in the section “Discussion” below.

## Discussion

The present study was performed to investigate if sex and brain ARs influence social preference and social memory, as well as the expression of 43 genes relevant for social behaviors in mice. Our results showed that, in contrast to male and female siblings, the AR^NesDel^ males lacked social memory when presented to male conspecifics, while all three groups displayed social preference and social memory when presented to female stimuli animals. In addition, an AR-independent sexual dimorphism was seen in relation to social investigation of female conspecifics whereas all three groups showed similar social interest toward male stimuli mice. Furthermore, object memory and olfaction were not affected in the AR^NesDel^ males. Our explorative study of genes relevant for social behaviors suggested expression differences between the three groups for the following genes: *Cyp19a1*, *Esr1*, *Oxtr*, *Cd38*, and *Ucn3* in the hypothalamus and *Cyp19a1*, *Cd38*, *Crh*, and *Gtf2i* in the amygdala.

Previous research have shown that male rodents tend to spend longer duration of social investigation than females (Johnston and File, 1991; Holmes et al., 2011; Dumais et al., 2013), and this has been suggested to be due to sex differences in testosterone levels (Thor, 1980; Thor et al., 1982; Tejada and Rissman, 2012). In line with these findings the male controls in the present study displayed more persistent social investigation of GDX stimulus females, and of odors from a cage with females, in comparison to female controls. This sexual dimorphism was, however, not seen when the test mice investigated male conspecifics in the sampling phase of the social discrimination test. Interestingly, our results show that a lack of ARs in the nervous system does not influence the persistence of investigation of females in the three-chambered apparatus test or of female odors in the olfaction test, or of conspecifics of either sex in the social discrimination task. The lack of importance of ARs on social investigation is rather surprising considering the marked reduction of sexual, territorial and aggressive behaviors reported from studies of males lacking ARs in the nervous system (Raskin et al., 2009, 2012; Juntti et al., 2010; Marie-Luce et al., 2013; Studer et al., 2015) or throughout the whole body (Sato et al., 2004). Our results are, however, similar to reports which show that AR^NesDel^ males show similar preference of females over males in various assays of mate preference behaviors (Raskin et al., 2009; Juntti et al., 2010; Marie-Luce et al., 2013). In contrast, a previous study of testicular feminized (Tfm) mice, carrying a naturally occurring disruptive *AR* mutation, have suggested major role for AR in partner preference (Bodo and Rissman, 2007). It may be speculated that these discrepancies may be due to differences in testosterone and estradiol levels between the AR^NesDel^ and Tfm males (Roof and Havens, 1992; Raskin et al., 2009), and to the fact that Tfm mice also lack ARs in the peripheral organs, which is in contrast to AR^NesDel^ mice.

Several studies show that sex steroid treatment may modulate social recognition (Bluthe et al., 1993; Bucci et al., 1995; Pierman et al., 2008; Spiteri and Agmo, 2009). In females, ERα, and to some extent ERβ, are crucial for short-term social memory (Choleris et al., 2003, 2006; Sanchez-Andrade and Kendrick, 2011). In males, ERα seems to influence long-term, but not short-term social recognition (Sanchez-Andrade and Kendrick, 2011). The role of ARs for social recognition has so far been sparsely investigated. In the present study we show that ARs in the nervous system of male mice are not necessary for social memory of female conspecifics whereas they are crucial for memorizing male stimulus animals. This result is difficult to interpret at this point, and needs to be confirmed and further explored in future studies. Intriguingly, however, Tfm mice also display alterations in social interactions dependent on the sex of the stimulus animals, which are not seen in sibling controls (Tejada and Rissman, 2012). In the light of our findings that oxytocin signaling (higher expression of *Oxtr* in hypothalamus and of *Cd38* in amygdala) is affected in AR^NesDel^ males compared to controls it is noteworthy that neuron-specific *Oxtr* knockouts (Nakajima et al., 2014) also display alterations in social interactions dependent on the sex of the stimulus animals. Furthermore, as testosterone inhibits long-term social recognition in male rats (Bluthe et al., 1993), we suggest that future studies investigate if testosterone and ARs modulate long-term social recognition in male mice. Even though ERα seems to play a crucial for social memory in female mice (Choleris et al., 2003), we also emphasize studies using homozygous AR^NesDel^ females to explore to what extent ARs may modulate social recognition also in female mice.

To better understand the results from the social memory tests, we performed NOR tests of the three groups. Both AR^NesDel^, as well as female and male control groups showed object memory, which may indicate that the reduced social memory seen in AR^NesDel^ males was not caused by a general memory deficiency. Our results are in line with those from (Rizk-Jackson et al., 2008) showing intact object memory in Tfm male mice lacking functional ARs. They, however, contrast results from rats showing that only intact and GDX male rats with testosterone, but not estrogen treatment, display object memory (Aubele et al., 2008). These differences may be due to differences between species and experimental designs. Both Tfm male mice and rats tend to spend less time exploring a novel object in an open field compared to wildtype male mice indicating elevated anxiety levels in rodents lacking ARs (Zuloaga et al., 2008a,b, 2011; Chen et al., 2014). We, however, did not see any genotype differences in object investigation time during the sampling part of the NOR test, which may be due to the considerable differences in experimental procedures and outcome measures.

The *Ar* expression was, as expected, strongly reduced in the hypothalamus and amygdala of AR^NesDel^ males compared to control males and females, verifying the functional *Ar* deletion in the knockout mice. To investigate to what extent expression levels of genes of known importance for social behaviors differ between the sexes and/or are modulated by AR, more than 40 genes were analyzed in the amygdala and the hypothalamus of the three groups. As sex steroids modulate social investigation and recognition, a substantial number of genes involved in the synthesis and function of estrogens and androgens were investigated (**Table 1**). Out of those *Cyp19a1*, encoding the aromatase enzyme, and *Esr1*, encoding the ERα, were differentially expressed. Intact males displayed higher aromatase expression than females in the hypothalamus, and higher levels of aromatase than AR^NesDel^ animals in the amygdala. This is to a large extent in line with previous reports describing a higher aromatase expression in most brain areas of males compared to females, which is due to regulatory effects of both ARs and ERs (Stanic et al., 2014). To what extent the lower expression of aromatase in amygdala of AR^NesDel^ males compared to control males may influence male recognition warrants further investigations. In hypothalamus, but not amygdala, *Esr1* expression was elevated in females compared to both male groups, which is supported by previous studies (Perrot-Sinal et al., 1996; Raskin et al., 2009).

The expression and function of the neuropeptides oxytocin and vasopressin and their receptors have been reported to be sexually dimorphic in various brain regions (Gabor et al., 2012; Dumais et al., 2013). These sexual dimorphisms are mainly due to the actions of sex steroids (Young, 2007; Bao and Swaab, 2010; Bourghardt et al., 2010). Although it is well-known that estrogens, through ERα and ERβ, regulate the amount of oxytocin in the hypothalamus and of the receptor expression in target areas (Choleris et al., 2006; Bao and Swaab, 2010), the role of AR in this context is sparsely investigated. Therefore it is interesting that we not only detected higher hypothalamic expression of *Oxtr* in females than in males, but also that ARs seem important for this difference. Furthermore, *Cd38*, recently shown to be an important regulator of oxytocin release as well as of social behaviors (Higashida et al., 2012), was expressed to a lower extent in the hypothalamus of females compared to both male groups. However, in the amygdala, where the role of CD38 for oxytocin function and social behaviors is less known, AR^NesDel^ males showed elevated *Cd38* expression compared to both male and female controls. These findings are novel and highly interesting, not least in relation to the hypothesis that oxytocin and androgens exert opposite effects on social–emotional behaviors in humans (Rood et al., 2008), and should hence be further evaluated in future experiments. In line with previous results, we did not reveal any significant alterations of mRNA levels of oxytocin or vasopressin when comparing mice lacking ARs with controls (Sato et al., 2004; Marie-Luce et al., 2013). Furthermore, regarding the well-established sexually dimorphic expression of vasopressin in medial amygdala and BNST (Rood et al., 2008) we did not detect any difference in the former nucleus and latter was not investigated in the current study. In contrast to others we did not see any alterations of nNOS expression in *Ar* knockout mice compared to controls (Cooke et al., 1993; Schmidt et al., 2000; Sato et al., 2004).

Out of the investigated genes involved in stress regulation, *Crh* and *Ucn3*, both influencing social memory in mice (Deussing et al., 2010; Camats Perna and Engelmann, 2015), showed sexually dimorphic expression. As recently reported, the amygdala expression of *Crh* was higher in males than in females (Sanchez-Andrade and Kendrick, 2009). This sex difference was independent of the presence of AR which supports previous studies showing increased *Crh* expression in amygdala after estrogen treatment (Jasnow et al., 2006). The *Ucn3* expression was lower in the hypothalamus of males than in females, again independently of the presence of ARs in the male brain. These sex differences may be of importance for the previously described sexually dimorphic actions of the CRH and urocortins on social (Spehr et al., 2006) and other behaviors.

Finally, the amygdala expression of *Gtf2i* was elevated in both females and AR^NesDel^ males compared to control males. This is interesting since this gene is located in the chromosomal deletion causing William’s syndrome, characterized by a hypersocial personality. Human genetic association studies (Steeb et al., 2014) and investigations of *Gtf2i*-deficient mice (Sakurai et al., 2011) suggest that *GTF2I* may be a major contributor to the hypersocial phenotype seen in William’s syndrome patients. Expression differences of *Gtf2i* may hence contribute to the effects of sex on social investigation and of ARs on male recognition observed in the current report.

Some limitations of our study should be considered. Previous studies report that intact AR^NesDel^ males display elevated levels of testosterone and estradiol compared to control siblings (Raskin et al., 2009). Therefore, it cannot be ruled out that some of our behavioral and gene expression results could be partly influenced by enhanced sex steroid actions through the ERs (since AR expression is lacking in the knockout siblings). In the current study AR deficiency was only investigated in male mice. Since ARs regulate social behaviors and brain functions also in female mice (Sato et al., 2004) future studies should investigate sociability, social recognition, and gene expression in homozygous AR^NesDel^ females. As the gene expression studies were conducted on tissue dissections comprising all nuclei of hypothalamus and amygdala, respectively, and these various nuclei, of which most contain sex steroid receptors, regulate various brain functions, strict interpretations of relations between specific expression differences and particular behaviors should be avoided.

## Conclusion

Our main results suggest that sociability and social memory are AR-independent abilities in males interacting with females whereas the presence of AR is crucial in order to recognize a male conspecific. Additionally, our findings indicate that ARs do not modulate social investigation, NOR or odor detection. Furthermore, genes related to oxytocin, estrogen and William’s syndrome seem to be regulated by ARs.

## Author Contributions

Study concept and design: SK, LW. Acquisition, analysis, or interpretation of data: SK, ES, PK, and LW. Drafting of the manuscript: SK, LW. Critical revision of the manuscript for important intellectual content: SK, ES, PK, and LW. Final approval of the version to be published: SK, ES, PK, and LW. Agreement to be accountable for all aspects of the work in ensuring that questions related to the accuracy or integrity of any part of the work are appropriately investigated and resolved: SK, ES, PK, and LW.

## Conflict of Interest Statement

The authors declare that the research was conducted in the absence of any commercial or financial relationships that could be construed as a potential conflict of interest.
